# A Theoretically-Sufficient and Computationally-Practical Technique for Deterministic Frequency Seriation

**DOI:** 10.1371/journal.pone.0124942

**Published:** 2015-04-29

**Authors:** Carl P. Lipo, Mark E. Madsen, Robert C. Dunnell

**Affiliations:** 1 Department of Anthropology and IIRMES, California State University Long Beach, 1250 Bellflower Blvd., Long Beach, CA 90840, USA; 2 Department of Anthropology, Box 353100, University of Washington, Seattle, WA 98195-3100, USA; 3 Department of Anthropology and Middle Eastern Cultures, P.O. Box AR, Mississippi State University, MS 39762, USA; Universidade do Algarve, PORTUGAL

## Abstract

Frequency seriation played a key role in the formation of archaeology as a discipline due to its ability to generate chronologies. Interest in its utility for exploring issues of contemporary interest beyond chronology, however, has been limited. This limitation is partly due to a lack of quantitative algorithms that can be used to build deterministic seriation solutions. When the number of assemblages becomes greater than just a handful, the resources required for evaluation of possible permutations easily outstrips available computing capacity. On the other hand, probabilistic approaches to creating seriations offer a computationally manageable alternative but rely upon a compressed description of the data to order assemblages. This compression removes the ability to use all of the features of our data to fit to the seriation model, obscuring violations of the model, and thus lessens our ability to understand the degree to which the resulting order is chronological, spatial, or a mixture. Recently, frequency seriation has been reconceived as a general method for studying the structure of cultural transmission through time and across space. The use of an evolution-based framework renews the potential for seriation but also calls for a computationally feasible algorithm that is capable of producing solutions under varying configurations, without manual trial and error fitting. Here, we introduce the Iterative Deterministic Seriation Solution (IDSS) for constructing frequency seriations, an algorithm that dramatically constrains the search for potential valid orders of assemblages. Our initial implementation of IDSS does not solve all the problems of seriation, but begins to moves towards a resolution of a long-standing problem in archaeology while opening up new avenues of research into the study of cultural relatedness. We demonstrate the utility of IDSS using late prehistoric decorated ceramics from the Mississippi River Valley. The results compare favorably to previous analyses but add new details into the structure of cultural transmission of these late prehistoric populations.

## Introduction

Seriation is a set of methods which use historical classes to chronologically order otherwise unordered archaeological assemblages and/or objects [1]. Frequency seriation is a technique that produces chronological sequences by arranging descriptions of assemblages so that the frequencies of artifact classes jointly form unimodal distributions. Developed in the early 20th century, frequency seriation played an integral role in the emergence of archaeology as a coherent discipline [2] and enabled culture historians to construct regional chronologies of prehistory throughout the New World [3–12]. Yet, for the last 50 years, frequency seriation has been largely ignored due to its association with relative chronology and the mistaken belief that radiometric dating techniques have replaced it. Saddled with a prevalent misunderstanding that seriation is simply a “dating method” [13] that is useful only when radiocarbon dating is impossible [14], seriation has never been fully developed as a computational algorithm. While there has been some interest in seriation for disciplines outside of archaeology [15–18], to the extent that methodological development has occurred in archaeology over the last 50 years, the focus has been largely on reducing the method to probabilistic similarity-ordering problems that can be attacked via multivariate statistical methods [19–23].

The roots of frequency seriation, however, stem from a deterministic algorithm that identifies orders on the basis of occurrence and frequency criteria. [1]. Recently, deterministic frequency seriation (hereafter, DFS) received some attention due to the demonstration that the method can be theoretically rationalized using an evolutionary framework. While the potential of this idea has been long recognized [24–26], the work of Neiman [27] firmly established an explanatory basis within cultural transmission models for the unimodal distributions that form the core of the frequency seriation algorithm. Neiman’s achievement has led to the re-imagining of DFS as a general tool for studying patterns of cultural inheritance within populations through time and across space [28–40]. With these advances, there remains substantial promise for DFS to again become a primary tool for archaeological analyses as it enables researchers to quantitatively track patterns of interaction, define social communities, and trace lineages among past populations, in addition to informing upon chronology. In this way, frequency seriation could serve as a key method in the establishment of a fully evolution-based discipline.

Despite its potential, the use of DFS as a productive tool for archaeological research remains difficult, and methods for constructing and evaluating solutions are incomplete. While a handful of assemblages can be seriated using hand manipulation, sorting through all possible orderings for a set of assemblages is neither feasible nor systematic. When the numbers of assemblages grows, a combinatorial explosion sets in, first visible once 10 or more assemblages are analyzed. The order of magnitude of numbers involved makes brute force approaches impossible even using modern computing power. This limitation was recognized early in the discipline. When archaeologists became concerned with the quantitative basis of their methods, probabilistic approaches were developed that could construct orders on the basis of similarity scores [41–49].

With probability-based seriation techniques one is guaranteed to find a solution, but the order produced reflects sources of variability beyond time including the effects of sample size, biased transmission processes and spatial variation [1]. While one may suspect that the final order is largely chronological, it is not possible to ascertain the degree to which the order represents time or other possible factors. The order of any particular subset of assemblages might be explained as a consequence of several factors: chronological order, layout in space, differences in the relative degree of contact between populations—or some combination of these factors. Allowing a computational method to obscure the causal influence of these factors destroys the value that seriation can have in helping disentagle such factors in real data sets.

Here, we introduce a new quantitative seriation algorithm that addresses the computational barrier inherent in DFS while also building upon the logical structure of the original method. The algorithm succeeds by iteratively constructing small seriation solutions and then using the successful solutions as the basis for creating larger ones. Significantly, the proposed algorithm produces the entire set of unique valid seriation solutions, and does not stop when a single valid solution has been located. This is important because there are typically a number of valid orderings. Some are suboptimal solutions because they are subsets of larger, more complete ones. Others are simply valid alternative solutions, which point to the influence of multiple causal factors. By including all valid orders, one can use the distribution of solutions as data regarding the structure of interaction between localities, and thus evidence about past cultural transmission. Our algorithm also enables statistical assessment of the significance of solutions, given the sample sizes employed. Using an example from the Mississippi River Valley, we demonstrate how the new algorithm provides detailed insight into the temporal and spatial structure of inheritance. Suitably extended in this way, we argue that DFS has the potential to inspire new innovative approaches to the archaeological record as much as it did in the 1930s as a critical tool for building chronology.

## Materials and Methods

### A Short History of Seriation in Archaeology

While not in common usage, seriate and seriation are English words that refer to arranging or occurring in one or more series [50]. The terms describe an archaeological method without defining it—there are many ways to order or arrange items in a series. The origins of the method are a bit opaque since variants were in used before it was given the name. Identifying its history and understanding the scope of the method, therefore, requires tracing the components involved in seriation that emerge over time and under which contemporary seriation now exists.

Sir Flinders Petrie [51] is generally credited with inventing seriation. Working with predynastic Egyptian materials, Petrie used ceramics found in graves to develop a chronology. Petrie’s break with archaeological tradition was to treat each grave lot as a sample of a continuous sequence of changing forms instead of as an exemplar of a period or stage. Since the history of Egyptian ceramics must have followed some particular course and thus presented an unique sequence of ceramic type replacements, the combinations of ceramic types found in grave lots allowed him to reconstruct both the history of ceramics and arrange the grave lots in chronological order. As in all seriation, the product was just an order; one had to determine independently (usually through superposition) which end of the order was most recent.

Alfred L. Kroeber [52] is credited with stimulating the American development. Kroeber did not cite Petrie’s work, and likely developed his version of seriation independently. The form and context of Kroeber’s proposal are dramatically different from Petrie’s and points strongly for an independent origin. Indeed, even in his seminal “Zuni Potsherds” (1916) paper Kroeber describes how the idea of extracting chronology from type composition occurred to him as he observed variability in pottery decoration among Southwestern pueblo deposits. The primitive seriation proposed by Kroeber was quickly amended by Leslie Spier, Alfred V. Kidder and Nels C. Nelson all of whom were conducting stratigraphic excavations in the American Southwest [7, 50, 52–54]. This group of researchers all noticed that when ceramics were described in a particular way—called “stylistic” by Kidder [7]—the temporal distribution of the types took the form of “normal curves.” Coupled with Kroeber’s initial insight, it was apparent that a series of assemblages collected from the surface or otherwise undated could be arranged in chronological order by rearranging them so that all type distributions approximated “normal curves” simultaneously.

As powerful as seriation proved to be, these early formulations were entirely intuitive and based on the generalization that greater temporal differences between assemblages caused larger differences between frequencies of decorated types. The shape of the curves that led to the ability to order assemblages were not justified and even the terms used were *ad hoc*: the distributions were not “normal” in a statistical sense. Since knowledge of rates of change was impossible, all that one could say about the characteristic distributions were that they were unimodal in that they had a single peak frequency and decreased in value away from the peak in both directions. Furthermore, there was little interest in figuring out why the characteristic distributions occurred. It was enough that they did and could be used to order assemblages. Rationalization was limited to rephrasing the frequency observations as “popularity,” and an answer to the question why did stylistic types display “normal distributions” was that styles simply increased in popularity until they reached a peak and then declined. Such statements are, of course, just descriptions of the observed frequencies and represent, moreover, the selection of simply one type of distribution that the popularity of styles can take. Seriation thus was based on an empirical generalization about the distribution of stylistic classes through time.

Almost all of the early work involved frequencies of stylistic (historical) pottery classes used as attributes of assemblages, the assemblages being groups of artifacts, usually but not always, pottery. But as Petrie’s work showed, the groups ordered might be objects, i.e., groups of attributes. Descriptions used for assemblages were frequencies of historical classes; those for objects were presence/absence tabulations. By the 1930s, use of the method had spread from the Southwest to include the Eastern United States and the Arctic and by the 1940s even Peru and Amazonia had chronologies based on seriation [9, 55]. James A. Ford [56, 57] played a critical role in disseminating the method so widely and was the only scholar to take an interest in the theoretical aspects of seriation until the 1970s [58–60]. Although Kroeber had been aware of potential problems derived from sample size effects, Ford brought these considerations to the fore, albeit in a highly intuitive, non-quantitative, and ultimately incorrect way. More importantly, he deduced a series of conditions under which the empirical generalization driving seriation might be expected to hold: (1) assemblages seriated must represent brief intervals of time; (2) assemblages seriated must come from the same cultural tradition; and (3) assemblages seriated must come from the same local area. The meaning of key terms like “brief interval,” “cultural tradition,” and “local area” were left undefined.

Ford, like his predecessor, arrived at the final arrangement by eyeballing trial and error orderings for conformance to the unimodal distribution model. Entirely a manual process, Ford’s technique requires arranging strips of paper representing assemblages and with type frequencies graphically depicted as bars. One would move the strips around until the pattern of the bars in each type would match “battleship-shaped” curves. For many workers, this crude process was a critical failure of Ford’s technique. In 1951, George Brainerd and Eugene Robinson proposed an entirely new technique for arriving at the order of groups [43, 61]. They devised a measure of similarity, since termed the Brainerd and Robinson Index of Agreement or simply the Brainerd and Robinson Coefficient, with which pairs of assemblages could be compared in terms of type composition. Thus described, they noted that in correct solutions the most similar assemblages were adjacent to one another; since this order was unique, groups could be chronologically ordered simply by arranging them so that the most similar units were adjacent. Brainerd and Robinson did this by rearranging rows and columns in a square matrix (each group is compared with every other group) of similarity coefficients; in a perfect solution, the magnitude of the similarity coefficients would decrease uniformly (monotonically) away from the diagonal of the matrix (the groups compared with themselves). Cowgill [62] developed a similarity-based approach for occurrence descriptions paralleling the techniques developed by Brainerd and Robinson for frequency descriptions.

**Fig 1:**
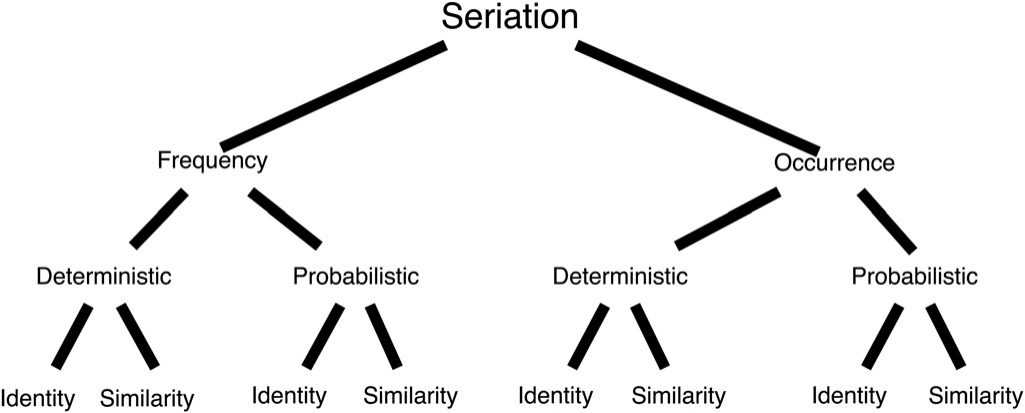
Classification of seriation techniques. Dunnell [63] defines seriation to be a set of methods which use historical classes to chronologically order otherwise unordered archaeological assemblages and/or objects. Historical classes are those which display more variability through time than through space. Occurrence seriation uses presence/absence data for each historical class from each assemblage [51, 52]. Frequency seriation uses ratio level abundance information (in percentage for) for historical classes [54, 57, 64]. Frequency and occurrence seriation techniques can take the form of deterministic algorithms that require an exact match with the unimodal model or probabilistic algorithms that accept departures from an exact fit. Identity approaches employ raw data (whether frequency or occurrence) to perform the ordering. Similarity approaches transform the raw data into a non-unique coefficient (e.g., Brainerd Robinson, squared Euclidean distance); the coefficients then form the basis for ordering.

Thus, two kinds of seriation approaches emerged. Occurrence seriation uses presence/absence data for each historical class from each assemblage [51, 52]. Frequency seriation uses ratio level abundance information for historical classes [54, 56, 57]. Like Ford, one could insist on an exact match with the unimodal model before regarding an order as chronological, a deterministic solution. Alternatively one could accept the “best fit” to the unimodal model as chronological, a probabilistic solution [63]. Each of these approaches to seriation can subsequently be built to utilize raw data (identity information whether frequency or occurrence values) or similarity coefficient (e.g., Brainerd Robinson, squared Euclidean distance) to form the basis for ordering. Thus, as shown in Fig 1 with two kinds of description (frequency/occurrence), two approaches to ordering (identity/similarity), and two possible solutions (deterministic/probabilistic), there are eight different families of seriation techniques available to archaeologists [1, 63]

Since Brainerd and Robinson [43, 61], the majority of efforts have focused on probabilistic approaches and researchers have brought increasingly sophisticated numerical approaches to bear on seriation [46, 65–76]. These probabilistic approaches generally seek to find approximate solutions by reducing the dimensionality of the data set. They will find a solution even when joint unimodality is not possible and most measure the departure from a perfect solution by calculating stress (residuals) or by examining variability within higher dimensions. As a whole, these techniques treat seriation as an empirical generalization about the way “data change” through time rather than a set of theoretical rules used for explanation. Variability in the frequencies of classes beyond the generalization is treated as noise rather than information about violations to the model and much of the utility of deterministic solutions that can be created by hand ordering is lost. Consequently, most of these quantitative approaches remain in the programmatic literature. Most practical work continues to be done pretty much as Ford did it in the 1950s, hand creating orders using graphical representations of relative frequencies in order to establish deterministic solutions.

### Explaining Seriation

To understand how to build an automated algorithm that is true to the seriation method, one must look in detail at its requirements. In his 1970 paper, Dunnell evaluated Ford’s criteria [1, 56, 57]. Ford’s conditions 1 and 2 were found to be sound and conditions that groups to be seriated (objects or assemblages of objects) had to meet for the generalization warranting the method to apply. Groups did not have to be of short duration (time between the addition of the first and last element to the group) in some absolute sense as Ford supposed, but group duration did have to be comparable among the included cases. Groups did have to belong to the same tradition (ancestor-descendant relationships). While there was no way to assess whether these conditions were met *a priori* by a given set of assemblages, Dunnell showed that when deterministic-identity approaches were used, seriation could not be made to yield incorrect answers on these grounds, thus securing the chronological warrant for arrangements derived by those techniques. The other techniques are not robust in this regard and the orders arrived by those means may or may not be chronological.

The “local area” criterion proved to be another matter. Dunnell [1] showed that this condition did not apply to the groups to be seriated as Ford had assumed. Rather it was a deficiency in the warranting generalization; the method was under determined. The generalization only spoke to temporal distributions of types, not their spatial distributions. As Ford intuitively appreciated and others showed empirically in the 1960s, frequencies of types varied in space and that variation could be mistaken for difference in age. Ford’s solution was to limit the amount of space in a seriation, but this was a heuristic and did not address the underlying issue. To get rid of spatial variations would limit a seriation to a simple point in space; one would simply be doing superposition under a different name. Using the different properties of space and time, Dunnell showed that the effect of spatial variation could be eliminated by multiple seriations of the same events using different materials (e.g., pottery types, point types, grave types, etc.) and extracting the common order as chronological. Seriation thus became a more complicated and demanding dating method. Archaeological reaction to this was mixed. Many simply abandoned the method relying on other methods like radiocarbon dating wherever possible; others simply ignored the limitations of seriation and continued in the manner of Ford.

### Limits of Current Seriation Approaches

Thus, it is not accidental that most practical approaches to creating deterministic seriation solutions have remained largely hand-built despite the availability of computer processing tools. Seriation, whether employing class frequencies or simple occurrence information to order assemblages, yields solutions that are identified from the permutations of the set of assemblages. The set of possible permutations that must be examined is vast in numbers. Moreover, seriation has been related to the “traveling salesman problem”(TSP) in which one is given a list of cities and their pairwise distances, and tasked to find the shortest possible route that visits each city exactly once and returns to the origin city [76–78]. If one tries to solve the TSP by examining all possible routes, it quickly becomes impossible as the number of solutions increases as the factorial of the number of cities in the list. Given the number of solutions that must be searched, even parallel clusters of the fastest available computers are insufficient when the number of assemblages gets larger than 14. As described in more detail by Madsen and Lipo [79], the problem is even worse than factorial, in that the best seriation solution may be a combination of sub-solutions which break the available assemblages into sets. When this possibility is included, the growth of possible solutions is even greater than factorial (Table 1).

**Table 1:** Number of total solutions with multiple seriation groups and processing time for sets of assemblages 4 < *N* < 100, testing solutions across a computing cluster with 64 cores, 5*μs* per evaluation. Once the number of assemblages is greater than 14, brute force methods requiring one to search all possible options clearly becomes impossible even with the fastest available computers working in parallel.

N	Total Solutions	Seconds	Years
4	15	0.00012	3.7e-12
6	4.7e+02	0.0037	1.2e-10
8	5.2e+04	0.4	1.3e-08
10	1.5e+07	1.1e+02	3.6e-06
12	8.5e+09	6.6e+04	0.0021
13	2.6e+11	2e+06	0.064
14	8.9e+12	7e+07	2.2
15	3.5e+14	2.8e+09	87
16	1.6e+16	1.2e+11	3.9e+03
20	1.7e+23	1.3e+18	4.2e+10
40	9e+65	7e+60	2.2e+53
60	5.1e+116	4e+111	1.3e+104
80	5.1e+172	4e+167	1.3e+160
100	4.4e+232	3.4e+227	1.1e+220

The combinatorial challenge with DFS has generally led many to use approximate approaches, based upon reduced similarity descriptions of type frequencies. Deterministic algorithms for frequency seriation, however, have advantages over similarity approaches since they make use of all of the type abundance information for each assemblage to build orders, thus allowing orders to be rejected and the search space thus reduced. Currently, hand-built approaches have been the only feasible way of creating deterministic seriation solutions [32, 33, 80]. In addition to integrating pairwise statistical evaluation for comparison of assemblages [32], manual solutions have the advantage of a general pattern recognition strategy that is inherent in our cognition. The disadvantage of hand-built solutions, even augmented by pairwise significance tests and bootstrap confidence intervals [32], is that investigators tend to stop when they find a valid solution given the effort involved. But a solution may be one of many possible, each representing potential information about change in cultural traits and their spatiotemporal histories. If what we seek is not merely a rough chronological order but information about cultural transmission, then we need to study all of the solutions.

Ultimately, neither manual sorting nor probabilistic methods are satisfactory since the strength of seriation as a method rests on statistical assessment of all solutions that match the dual requirements of continuity and unimodality. Thus, an exhaustive characterization of the search space to find all of the valid orders is integral to the method. In addition, we need to know how sets of assemblages fail to produce a valid seriation order. Since we explain variability in frequencies as a function of transmission through time and space, finding the points at which assemblages cannot be fitted together is as important as finding those assemblages that can be seriated [32, 80]. In contrast, probabilistic orderings force all data points into a single solution, and thus are limited in their ability to locate the boundaries at which seriation solutions cannot be constructed. As a consequence, probabilistic seriation methods are generally unsuitable for disentangling the contributions of space, intensity of contact, and time.

**Fig 2:**
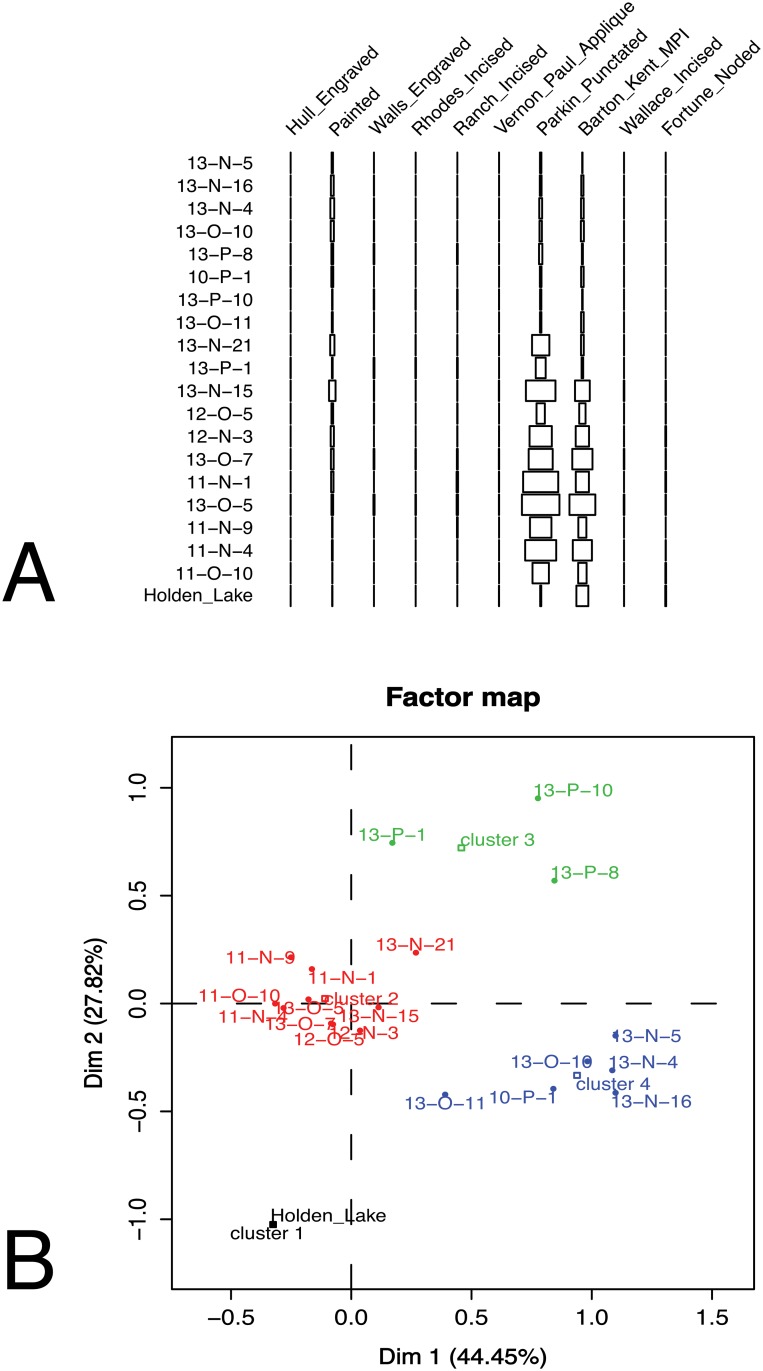
The results of a probabilistic seriation analysis for a set of late prehistoric ceramics assemblages from the Memphis and St. Francis areas as described by Lipo [84] and Phillips and colleagues [10]. Here, the figures show the the results of correspondence analysis (CA) for the dataset in Table 2 following [85]. (A) The seriation order produced from the CA shown in standard centered bar format. (B) CA results shown with clusters as determined by hierarchical cluster analysis on the principle components. One can see that the change in the frequencies of types roughly follows a unimodal distribution, but there are numerous violations of unimodality as well. Data and R code for the correspondence analysis are available at https://github.com/mmadsen/lipomadsen2015-idss-seriation-paper.

**Fig 3:**
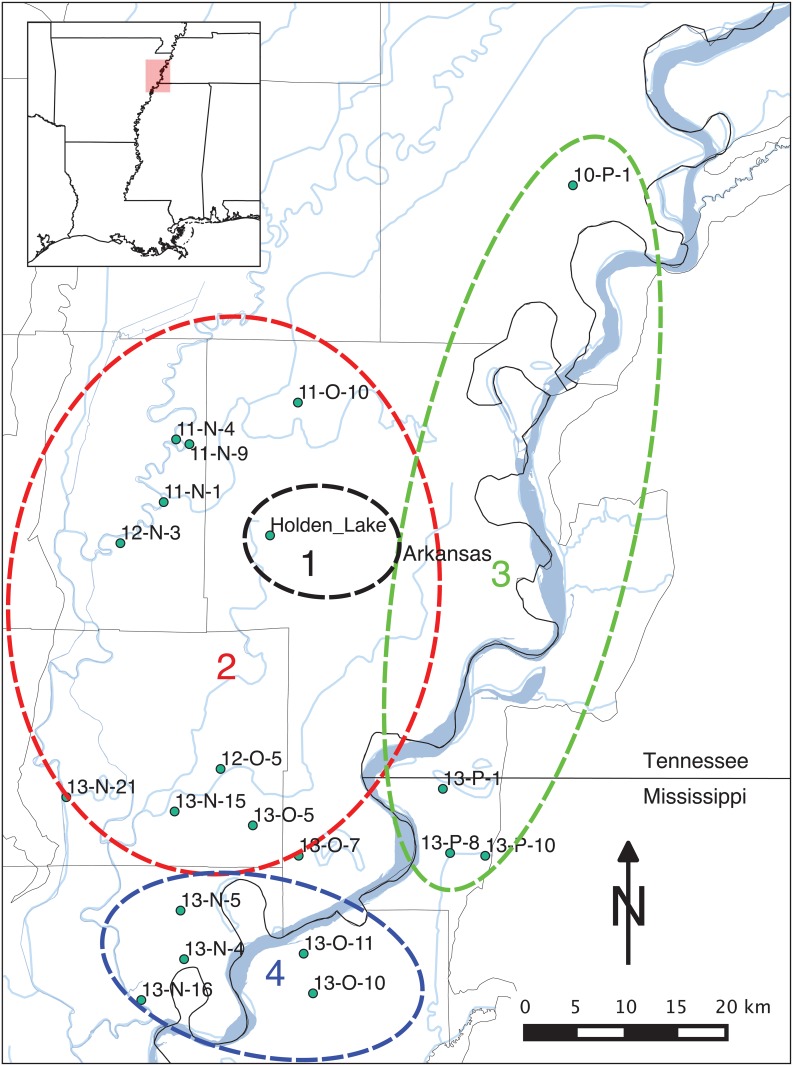
Spatial groups of assemblages as determined by the hierarchical cluster analysis of the principle components generated through the CA analysis as shown in Fig 2.

Fig 2, for example, demonstrates the kind of results that occur using correspondence analysis, which is the best available probability-based seriation technique [39, 69, 81, 82]. The example is a set of assemblages of well-described ceramics from the lower Mississippi River Valley [10, 32, 83]. As shown in Panel A of Fig 2, the results generally meet the expectation of unimodality, but there are many deviations in the distribution. When we examine the distribution of the assemblages that comprise the solution (Fig 2, Panel B and Fig 3), we can see that the type frequencies show substantial spatial patterning. The problem, however, is that given any order, how does one distinguish the varying effects of space from those of time? How does one trace the population structure separately from both time and space?

### A Model-based Approach To Solving The Seriation Conundrum: IDSS

We argue above that seriation would be greatly improved by returning to deterministic seriation methods that use identity data. We further contend that basing seriation algorithms on the behavior of cultural transmission models derived from evolutionary theory will reduce the scope of the seriation problem, by giving us specific patterns to search for and thus winnowing candidate solutions more strongly than do methods which employ similarity data. Dunnell [26] showed that evolutionary theory can explain why the empirical generalizations driving seriation are true (to the extent they are) and when they fail. Taking historical classes to represent neutral traits (i.e., traits that have no measurable differences in terms of performance and/or cost), the forces that primarily act on their temporal and spatial distribution are stochastic (drift). This is what produced both the unique, historically non-repetitive sequence of forms on which the seriation method depended and also accounted for unimodal distributions of relative abundances. Others have extended this work considerably [27, 32, 34, 40, 80, 83].

While Neiman [27] has shown that cultural transmission of neutral traits does not always produce unimodal distributions, those distributions of class frequencies that are unimodal have a significant chance of being the product of cultural transmission. Further exploration of the relation of unimodality and culture historical practice is warranted but beyond the scope of this paper. When it occurs, however, joint unimodality across several classes is a unique marker which is exceedingly unlikely to occur by chance and definitely occurs through the spatiotemporal diffusion of traits within an interacting population. Thus, where it occurs, unimodality and especially the joint unimodality of multiple classes is a much stricter criterion to use in constructing seriation solutions than monotonic ordering of similarity indices. Many fewer candidate solutions will display joint unimodality than do monotonic similarity, and thus the use of joint unimodality helps avoid the need for brute force enumeration of possible solutions, given an appropriate search method.

In addition, cultural transmission models describe the flow of traits as having continuity within the limits of sampling and population size. In other words, we do not expect large jumps or discontinuities, and can use this criterion as a way of ranking possible solutions and even eliminating candidates that display large gaps in frequencies but otherwise are unimodal. Employing both continuity and unimodality as patterns or criteria places very strong constraints on possible solutions, potentially reducing the number of candidates that must be checked by many orders of magnitude. In the following sections, we develop this intuition into an algorithm. That algorithm must meet several requirements in order to be useful.

First, the algorithm must allow the analyst to address all of the requirements of the seriation method including unimodality and continuity. Consistent with the practice of culture history in archaeology, we treat unimodality as a construct that serves with continuity to help identify patterns that are potentially the product of cultural transmission. Second, generation of candidate solutions should be automated, so that seriation can be used as part of larger analyses (e.g., spatial analysis, simulation studies of migration, trade, or cultural transmission). Third, the algorithm should provide error estimates and confidence bands where possible, to allow evaluation of the quality of a solution given the input data, and diagnosis of any violations of unimodality or continuity. Finally, the technique must be able to find all viable deterministic solutions given bounded and reasonable processing time for even relatively large sets of assemblage (e.g., 20 or 50), such that resampling or the bootstrap can be used to calculate error terms and evaluate the effects of sample size.

These are not easy requirements to meet. In the space created by all the possible orderings of assemblages, the vast majority of orders are invalid, as the combinations violate the conditions of the DFS method due to deviations from unimodality and/or continuity. Even with stricter constraints on possible solutions, valid candidates cannot be found by enumeration for more than a handful of assemblages. The search space must be “pruned” in some fashion to remove combinations that cannot possibly be part of a valid solution.

#### Overview of the IDSS Algorithm

**Fig 4:**
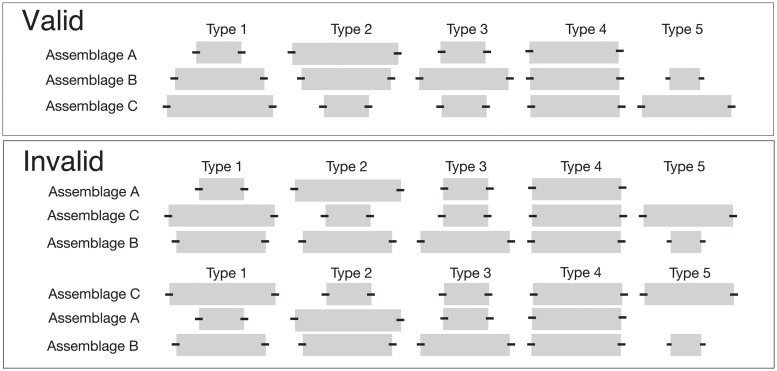
In DFS, assemblages must meet the frequency and continuity expectations of the model. Here, three assemblages (Assemblage A, Assemblage B, Assemblage C) are represented by rows of horizontal bars where the length of the bar is equivalent to the relative proportion of the type in the assemblage. The small black bars reflect statistical uncertainty of the proportions. At least three assemblages are required to evaluate orders based on the seriation model. Valid orders include type frequencies that include no change, types increasing in frequency, types decreasing in frequency, and types that have a single maximum frequency peak. Invalid orders are those with discontinuity in frequencies, those with more than one maximum frequency peak or in which the frequencies of types are increasing towards the top and bottom of the orders.

The technique we propose to accomplish these goals is called the Iterative Deterministic Seriation Solution (IDSS). IDSS builds DFS orders in an iterative process, starting with valid seriation solutions composed of the smallest possible number of assemblages and then employing these as building blocks for larger solutions. Solutions are grown from valid smaller solutions instead of enumerating possible combinations. We start with combinations of three assemblages (triples), the fewest number that can be evaluated in terms of the degree to which they meet the demands of the model. With three assemblages, we retain only those sets in which the frequencies for each of the classes show a steady increase, steady decrease, a middle “peak,” or no change at all (Fig 4). Assemblage orders that have frequencies that decrease then increase are eliminated as building blocks.

The next step in the procedure is to use just the successful triples and see if any of the remaining assemblages can be added to either end to create a larger set of four assemblages while also avoiding violations of the seriation model. The successful sets of four assemblages are then used to assess potential combinations of five assemblages, successful sets of five assemblages become the basis for looking at valid sets of six assemblages, and so on. This process is iteratively repeated until no additional larger seriation solutions can be validly created. The end product of this stage of the algorithm is the set of all valid seriation orders with the possibility that some assemblages may appear in more than one ordering.

The logical basis of this procedure is that all larger solutions consist of, by definition, smaller subsets of valid solutions. For example, a valid solution set of six assemblages labeled A-B-C-D-E-F also includes valid subsets such as B-C-D and B-C-D-E. Thus, if we start with valid solutions of N assemblages and iteratively evaluate N+1 assemblages in terms of the requirements of the seriation model, we are guaranteed to end up with the largest possible solution. Since the algorithm avoids having to search all of the combinations that stem from invalid solutions, IDSS vastly trims down the number of possible solutions: the search space is pruned as the algorithm proceeds.

While this iterative approach reduces the numbers of combinations, the numbers of possibilities that must be examined is still very large. While many of of these combinations are ultimately trivial since they often become parts of larger orders, when one is constructing solutions by aggregation, the smaller subsets must be searched before the larger seriation order is discovered. Nonetheless, building solutions by iterative “agglomeration” of smaller building blocks reduces the search space considerably, and by itself is enough to allow the analysis of reasonably sized and archaeologically-relevant data sets.

Scaling the algorithm to larger numbers of assemblages requires additional heuristics to further restrict the possibilities that must be evaluated. Solving this secondary problem requires further application of the theory underlying the seriation method. Ford’s [6] criterion states that for assemblages to be seriated, they must come from the same cultural tradition (see also [1]). This criterion means that the differences in frequencies between any two assemblages can be assumed to be a function of differences in the degree of interaction. In an ideal set of assemblages that reflect a single cultural tradition one would expect smoothly continuous frequency changes. When multiple cultural traditions are combined, the differences in frequencies will be discontinuous when considered as a group. What this means in practice is that relative discontinuities in frequencies potentially indicate the presence of more than one cultural tradition or that the changes in frequencies cannot be distinguished from sampling error. Resolution of these options potentially requires finding additional intermediate samples.

We can use the same continuity principle to rule out valid subset solutions that we do not need to evaluate. For example, A-D-G is a valid but trivial subset of the solution A-B-C-D-E-F-G. The differences in type frequencies of these subset solutions will be larger than the larger set. By assigning a threshold of discontinuity measured by the maximum allowable difference between the summed frequencies of any pair of assemblages within an ordered set, one can rule out most of the trivial solutions. Consequently, as we iteratively search for possible assemblages that can be added to either end of an existing one, we can rule out all of the possibilities that are too dissimilar for consideration. This step removes comparisons between assemblages and thus reduces our search space.

Of course, establishing a continuity threshold requires user input, which means that the search space is partially shaped by the researcher. However, this is always the case as we must select assemblages to include in our analyses. In the traditional practice of culture historians, this was accomplished by selecting those assemblages that come from a local area and that appear to come from the same cultural tradition [1]. In IDSS, we make this step explicit and thus amenable to automation and statistical evaluation by specifying the maximum discontinuity allowable within a set of assemblages that can be considered as being directly related to one another. In practice, this means stipulating a maximum frequency difference in any one type or the maximum allowable for the sum of frequency differences across all types. In an ideally generated set of assemblages that provides a good sample of the interacting population, the greatest difference between the frequencies of types would be relatively small (e.g., 5% or smaller) since good sampling should ensure continuous change in frequencies. The size of the threshold in many cases will be a reflection of the degree to which the assemblages are samples of the set of events that produced the assemblages in the first place. In most cases, the continuity threshold can be set higher to tolerate bigger gaps in the frequencies, but at the cost of a greater amount of processing required to search for solutions. The optimal value of the continuity threshold may also be determined algorithmically by repeating the analysis across several threshold values and examining how the structure of solutions change.

#### Initial Implementation

**Fig 5:**
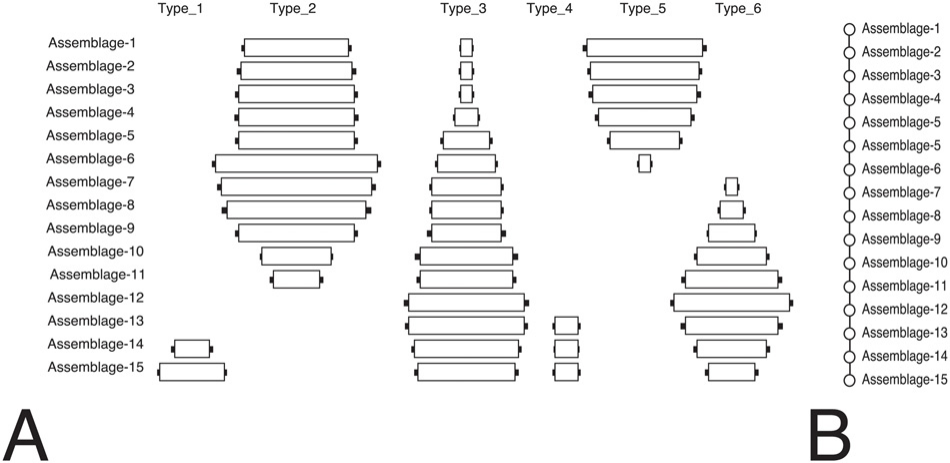
Example of the IDSS algorithm seriation output for 15 assemblages with 6 types. This seriation order was generated using a threshold of 0.10. Panel A takes the form of traditional centered-bar format where the empty bars indicate relative proportion of the type and small black bars represent confidence intervals of *α* = 0.05 for the type proportions. Panel B is the same order in graph form. Note that while hand-sorting of this example data could be relatively easily done, the IDSS algorithm ensures that the longest possible solution or set of solutions is found. In real-world cases, patterns of type frequencies often includes the effects of sample size, space and other transmission processes. In these cases, a systematic deterministic method is necessary to ensure comprehensive and statistically sound solutions.

We have coded the IDSS algorithm in Python (see S1 Text for the full algorithm). Tests of our IDSS implementation show that with artificially generated data in which an *a priori* solution is known, the correct solution set is always identified. In Panel A of Fig 5, we show a set of 15 unordered assemblages each with 6 types. Using a threshold of 0.10 (i.e., the maximum acceptable difference between assemblage frequencies is no more than 10%), the IDSS algorithm was able to locate the optimal seriation order of these assemblages out of all possible valid solutions in just over 1470 seconds, using a 2013-era quad-core computer. This length of time might appear slow relative to quick hand-sorting but the results of intuitive shuffling of graphical representations cannot ensure that the largest possible order is identified nor can it find all the equally valid solutions that might be present. Traditional brute force sorting approaches that evaluate the entire search space can easily take many years (Table 1). Using IDSS instead of hand-sorting allows identification of all valid solutions from groups of 20 or fewer assemblages on a single desktop computer. Twenty or so assemblages is a common scale of analysis, at least for many archaeological cases conducted within local regions, and it is important for a DFS algorithm to be able to produce optimal solutions for this scale of data, on commonly available hardware. In particular, many large sets of assemblages break down into much smaller subsets when ordered and thus can be analyzed quickly. Solutions with larger numbers of assemblages or few solution subsets require carefully setting the maximum differences between assemblages and using a computing cluster to further parallelize the evaluation of solutions.

#### Graphical Representation

Fig 5, Panel A represents the traditional graphic form for seriations in which the width of the horizontal bars represents the magnitude of the frequencies of types for individual assemblages. This “stacked and centered bar” format is instantly recognizable by archaeologists, and is excellent for displaying the results of a seriation if there is only one causal factor driving the ordering (typically, time) and if no assemblages participate in more than one seriation. When the situation becomes any more complex than a single chronological order, we need a better visual representation.

Graphs, a collection of vertices and edges, provide an alternative means of visualization that accommodate linear orderings as well as more complex relations [86–90]. We can create a graph representation of our seriation results by connecting assemblages via edges in the sequence produced by the IDSS algorithm (Panel B, Fig 4). This simple graphic informs us about the relations between assemblages without the addition of the information regarding the composition of the types. The graph representation has an advantage over traditional centered-bar diagrams since it allows us to examine relations where assemblages may be shared in multiple valid solutions [89, 91].

**Fig 6:**
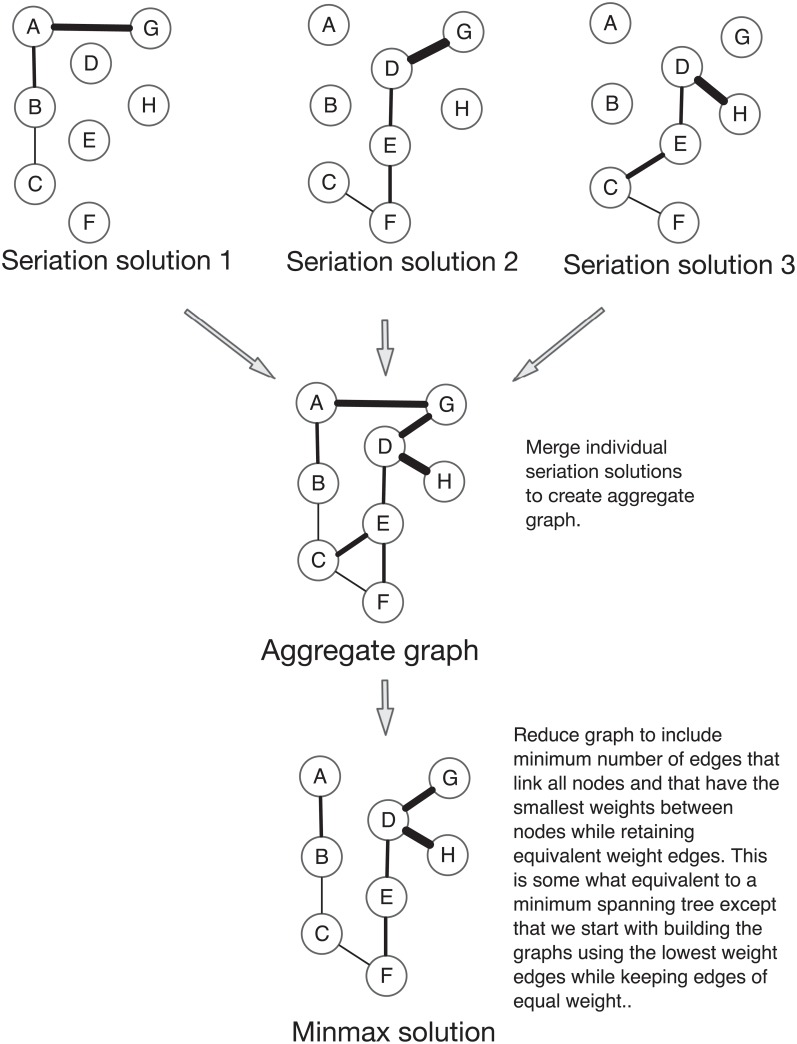
“Minmax” graph creation steps. In this example, we begin with the graph representations of three valid seriation solutions (1–3) for a set of 9 assemblages (A-I). In the figure, the thickness of the edges reflects the summed differences in frequencies between each pair of assemblages. Each solution represents a valid and unique seriation. To combine the three seriations, we first sum the graphs to create a single aggregate solution that is composed of all nodes and edges from the individual graphs. Using the aggregate solution, we then reduce the graph by including the fewest edges that can be made between all vertices and starting with the edges that have the smallest weight, as calculated by the sum of the differences in frequencies. Edges that include new vertices are added sequentially until all of the connected vertices are included. Edges with equivalent weight values are retained as well.

The ability of graphs to reflect complex sets of relationships, however, can result in difficult interpretation of the results. The strength of seriation is that solutions are linear relations where the order reflects some combination of differences in time and space. However, if assemblages are found in more than one solution, additional analytic steps must be taken to reduce the results to something that can serve as a hypothesis about the structure of transmission and the relations between assemblages. As shown in Fig 6, we can proceed by accumulating valid solutions, and then pruning unnecessary edges. We begin at the top with three valid solutions output from the basic IDSS algorithm. Each meets the criteria for unimodality and the frequency differences are within the 0.1 tolerance limit set for continuity. In the middle of the figure, we show the results of agglomerating the graphs together, where an edge exists between two vertices if those vertices possess an edge in any of the three source graphs. The weights assigned to edges are proportional to the summed differences in type frequencies between pairs of assemblages. This aggregate graph allows us to construct the final solution. We follow the approach described by Lipo [89], starting with just the vertices, and iteratively adding edges from the summed graph starting with those which possess the lowest weight as measured by Euclidean distance between pairs of assemblages. This process produces a graph that includes the maximal set of vertices from the starting solutions but using the minimum number of edges that represent smallest distances between vertices and includes all equivalent values as options. The result is what we call the “minmax” graph.

**Fig 7:**
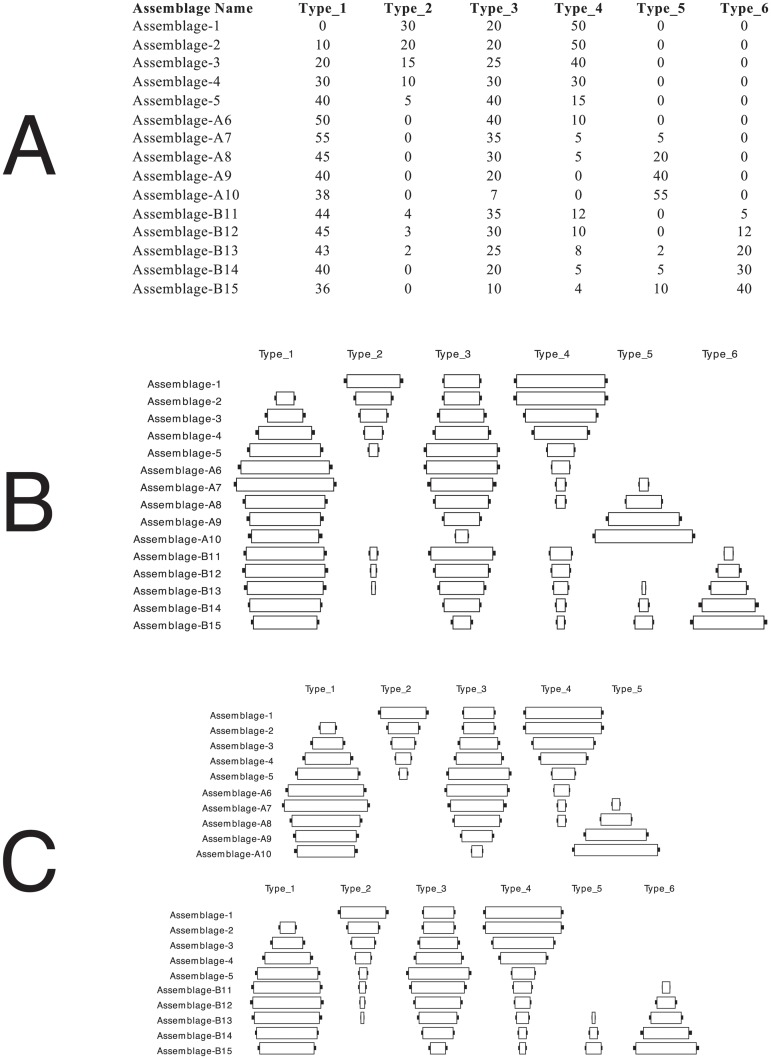
A set of assemblages that illustrate branching lineage. (A) Raw data for 15 assemblages with 6 types. (B) Centered bar graphical representation of the relative proportions of types for the 15 assemblages with confidence interval of *α* = 0.05. In this example, we can create valid DFS solutions that include all of the Assemblages 1–5 plus either the “-A” assemblages or the “-B” assemblages, but not both. (C) Seriation representation of the two lineages that make up the set of assemblages. Although they overlap for Assemblages 1 through 5, the two seriations cannot be merged into a single valid solution, and thus are shown in bar form as two separate solutions.

As an example, Fig 7 represents a simulated case in which a set of assemblages that initially represent a single lineage with a single temporal order branches into two sub-populations, each having valid seriation orders. Such a scenario might happen, for example, if a group of individuals who begin by exchanging information later becomes two distinct but smaller populations that only interact locally, or when a single location serves as a center node for two or more relatively separate sub-populations. In this scenario, there are 8 possible valid seriation solutions. Using a graph representation and the process described above, we can easily identify a pattern of relations in which the seriation branches into two different paths. The seriation solution we generate represents the minimum set of weighted edges which capture the smallest “weighted distance” between vertices. It represents, in this way, the minimal hypothesis about intensity of transmission and trait sharing needed to account for the observed pattern of frequencies.

#### Statistical Evaluation

In generating valid seriations that reflect variability in the archaeological record related to inheritance, we assume that the assemblages are described with three or more stylistic classes [25, 33] to avoid problems of closed arrays [2, 92–95]. We also assume that the assemblages have been evaluated in terms of minimum sample size requirements. Sample sizes must be great enough to ensure a minimum of statistical confidence in the frequencies of classes. In cases where samples are insufficient, the frequencies may reflect a lack of proper sampling and not the character of the archaeological record. Early culture historians used a fixed number such as 50 to be the minimum size required [10]. Bootstrap tests are a more robust means of assessing when samples are large enough to meet a specified statistical confidence level [32, 96].

Even when minimum sample size requirements are met, the comparisons between any pair of assemblages must be evaluated in terms of statistical reliability. The larger the sample size, the greater the confidence one has that the patterns between the frequencies of classes reflects the archaeological record and not the happenstance configuration of the sample’s description or other circumstances. This uncertainty propagates through the entire seriation order: all solutions obtained have statistical confidence based on the overall strength of the pattern between the pairs of assemblages.

To specify the statistical confidence of our seriation solution, we can construct confidence limits for the frequencies of individual classes. These confidence intervals then serve as the basis for assessing the strength of the pattern of frequencies. In terms of statistical models, a set of proportions from M classes is a sample from a multinomial distribution with M categories. Calculating confidence intervals for multinomial proportions is remarkably complex and there is not an exact method that is generally recognized. When the number of classes is “large” (i.e., *M* > 10), the Glaz and Sison [97] method is generally thought to be the best, while *M* < 10, Goodman’s method [98] is preferred. Since assemblages can vary in how many classes are represented, a better method is to use a bootstrap means for calculating the values for the bootstrap confidence intervals at a requested significance level for each pair of assemblages. This step consists of creating a large number of new bootstrap assemblages with the same sample size by resampling the original assemblage with replacement. In our implementation of IDSS, we calculate class frequencies for each of the bootstrapped assemblages. Using the pool of assemblages as the basis for the distribution of frequencies, we then determine the limits of the confidence intervals for the designated level of significance (*α*).

**Fig 8:**
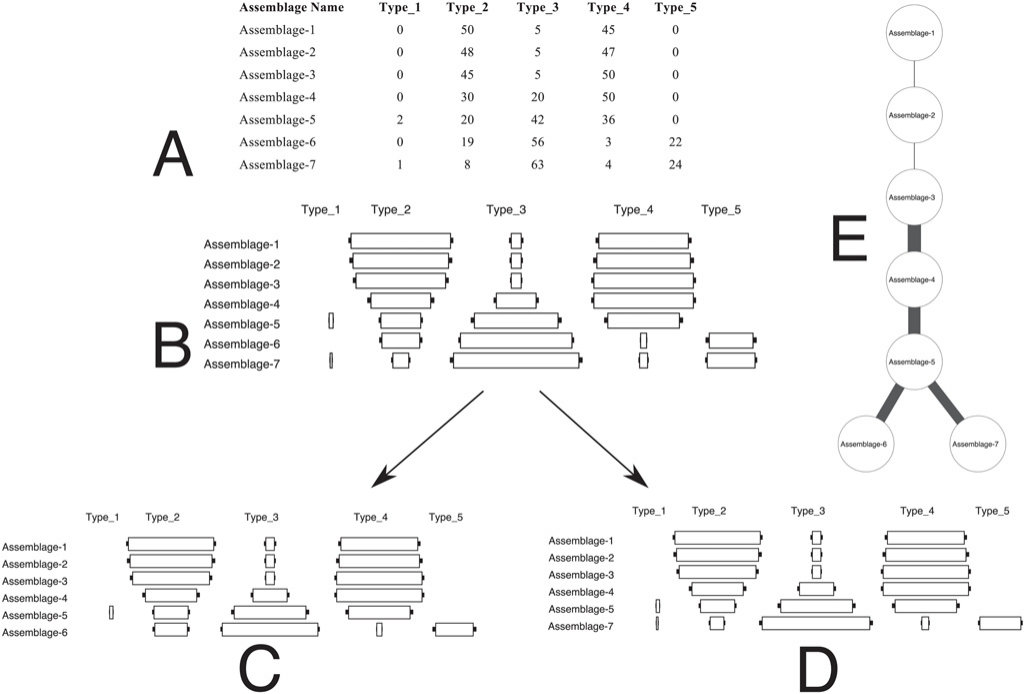
In A and B, 7 assemblages composed of material with 5 types are shown with a violation in the continuous distribution of frequencies. Comparing frequencies between assemblages relative to the DFS seriation model with a specified confidence interval of *α* = 0.001 and the bootstrap process described above, two valid solutions must be formed (C and D). These two solutions share Assemblages 1–5 but differ as to whether they include Assemblage-6 or Assemblage-7. (E) shows the two overlapping solutions in graph form.

We can then use bootstrap confidence limits when we make comparisons of frequencies between assemblages during the iterative testing steps. The differences between frequency classes must exceed the limits of the confidence interval in order for the pairs of assemblages to be evaluated having frequencies as “greater than” or “less than” one another. All comparisons in which frequency values fall within the confidence intervals are scored as “matching.” Since matching frequencies do not violate the assumptions of the frequency seriation model, this process has the effect of creating a greater number of solutions all of which are statistically valid orders at a given level of significance. Fig 8 provides an example of how bootstrapped confidence intervals can produce different solutions than using direct frequency comparisons especially when sample sizes of the assemblages or differences in frequencies being compared are small.

## Results

### Example From Phillips, Ford And Griffin (1951) And Lipo (2001)

Archaeological research conducted in the Lower Mississippi Valley (LMV) provides a useful example of how the concepts behind cultural transmission form the basis for generating explanations of the archaeological record, and no better case study exists than the long-term efforts of Phillips and his colleagues [10]. Through a series of surface collections of decorated prehistoric ceramics and the use of seriation to order assemblages through time, their work produced a solid chronological framework for the Mississippi River valley and established the region as a primary focus of American archaeology [2, 59, 99].

Using a subset of data from the LMV assemblages and new ceramic collections from seven deposits in northeastern Arkansas [32, 83] and shown in Table 2, Lipo used seriation-based techniques and simulations of cultural transmission to account for patterns of stylistic similarity in varying spatial and temporal configurations among 20 late prehistoric locations. Through his analysis, Lipo [80, 83] demonstrated that data generated from the original collections are well suited for examining transmission.

**Table 2:** Late prehistoric decorated ceramic assemblages from the Memphis and St. Francis areas of the Mississippi River Valley as described by Lipo [84] and Phillips et al. [10]. Analyses by Lipo [84] demonstrate that these assemblages have adequate sample size, classification consistency, no sherd size effects, and that the depositional environments are approximately equivalent. Given these analyses, we have confidence that the relative frequencies of ceramic types reflect patterns in the archaeological record and not the procedures involved in collection and description.

	Parkin Punctate	Barton/Kent/MPI	Painted	Fortune Noded	Ranch Incised	Walls Engraved	Wallace Incised	Rhodes Incised	Vernon Paul Applique	Hull Engraved
10-P-1	39	62	46	0	0	0	0	0	0	6
11-N-9	528	198	13	0	19	0	0	0	0	0
11-N-1	865	323	59	17	35	0	0	0	4	0
11-O-10	404	208	6	16	4	0	0	0	0	0
11-N-4	764	470	18	5	9	0	0	0	0	0
13-N-5	35	11	33	0	0	0	0	0	0	0
13-N-4	71	67	96	0	3	4	0	0	0	0
13-N-16	42	56	69	0	1	3	0	0	0	0
13-O-11	35	65	24	0	0	2	0	1	0	1
13-O-10	61	74	79	0	2	8	0	2	0	0
13-P-1	244	40	18	1	16	21	0	14	0	6
13-P-8	83	25	43	0	18	17	0	3	0	3
13-P-10	30	15	12	0	12	12	0	7	2	1
13-O-7	590	498	67	10	21	19	12	8	7	1
13-O-5	923	637	42	12	33	27	15	13	5	2
13-N-21	426	69	105	4	4	0	1	4	1	0
12-O-5	204	156	42	7	8	4	2	1	0	0
Holden Lake	27	294	7	24	2	0	2	1	3	0
13-N-15	728	364	160	9	5	8	14	3	7	2
12-N-3	549	328	77	19	4	0	3	1	2	0

**Fig 9:**
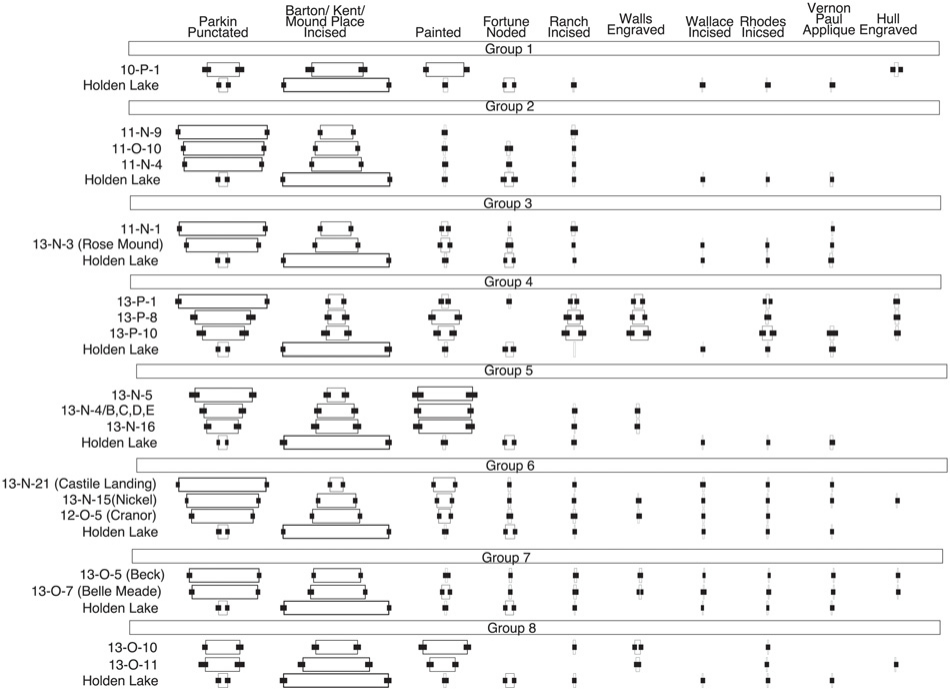
The set of DFS solutions created by hand sorting late prehistoric ceramic assemblages in the Memphis and St. Francis areas of the Lower Mississippi River Valley Survey [10, 83]. Here, the assemblages have been standardized in terms of type descriptions and are all of sufficient sample sizes. The error bars indicate the 99% confidence interval for the type frequencies. The largest seriation solutions formed eight spatial sets. The assemblage from Parkin (11-N-1) falls into two different sets, suggesting that it served as a central node of interaction between communities. The Holden Lake assemblage appears as a valid addition to all solutions, supporting the idea that it is earlier than the other samples in the analysis.

**Fig 10:**
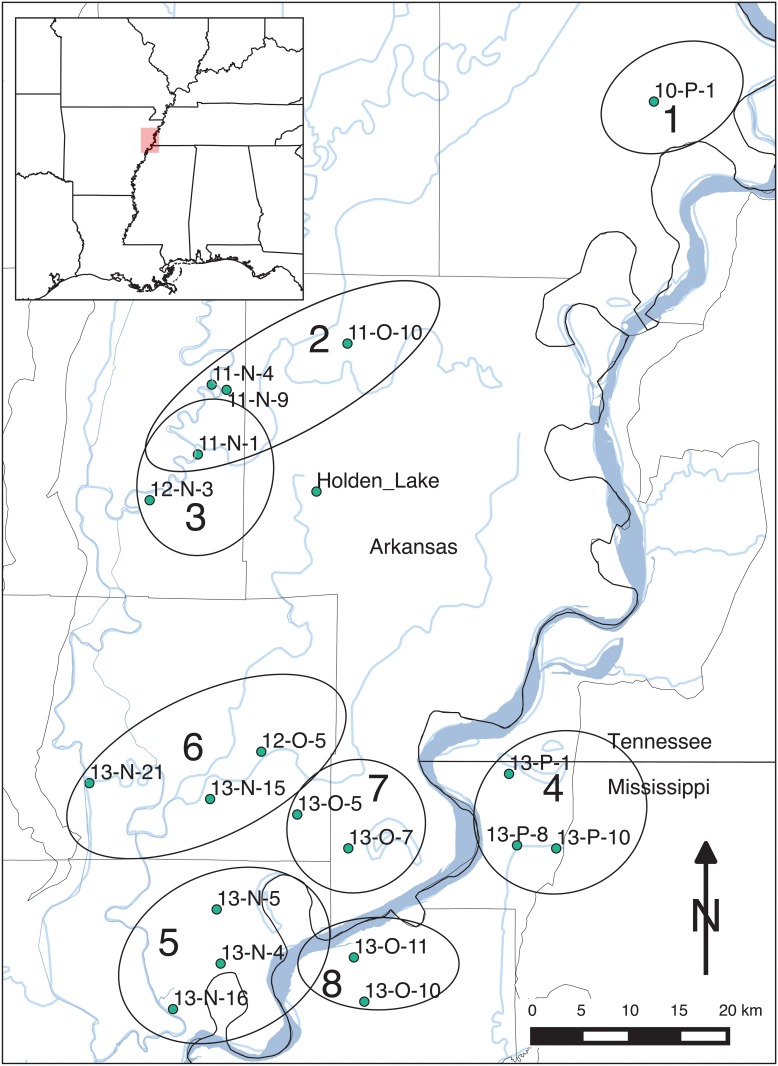
Spatial distribution of seriation groups with St. Francis and Memphis Assemblages consisting of Lipo [84] and Phillips et al. [10] samples. Labels for groups refer to seriation solutions numbered in Fig 9. While each seriation group also includes Holden Lake, this assemblage is removed here for visual clarity. The groups are strongly spatial in their configuration. Interestingly, the Parkin (11-N-1) assemblage falls into groups 2 and 3 suggesting that it served as a central node, possibly indicating emerging social complexity.

In his analysis, Lipo [80, 83] constructed deterministic seriations for the assemblages using a manual graphical technique and found that no single solution could be obtained using the 20 assemblages. Instead, the set of assemblages had to divided into 8 different spatial groups (Figs 9 and 10). These groups reflected the effects of local transmission among communities that overwhelms the effects of longer-range interaction within the region. Interestingly, two valid seriation solutions in the “Parkin” area (Groups 2 and 3 in Fig 10) overlap with one another in that they both share the assemblage 11-N-1, the Parkin site. Lipo [83] explained this result as the effect of Parkin acting as a central “node” in a network and possibly indicative of emerging social complexity among otherwise functionally redundant settlements.

While Lipo’s result demonstrates the potential for seriation as a means of explaining patterns of cultural transmission, the results and the approach as a whole are limited in practical utility for several reasons. First, the seriation results were created by hand sorting following graphic methods outlined by Ford [6, 10] though assisted using spreadsheet macros in Microsoft Excel [83]. Consequently, we have no way of knowing whether the final sets of orders are the largest set or whether all possible solutions are represented. Second, while Lipo created confidence intervals for each class frequency and tested the pairwise ordering of assemblages, the inability to assess the chosen solution with respect to the entire search space limits confidence in the results. Finally, the use of frequency graphs as the graphical representation for the set of solutions reveals the limitation of the visualization. Lipo showed that seriation orders overlapped or intersected with one another and that this overlap potentially allows one to infer information about prehistoric social structure, but the use of stacked and centered bar charts prevents effectively visualizing such relations. This limitation impacts the degree to which the approach can be systematically applied, especially as cases become increasingly complex.

### IDSS Analysis of PFG Assemblages

**Fig 11:**
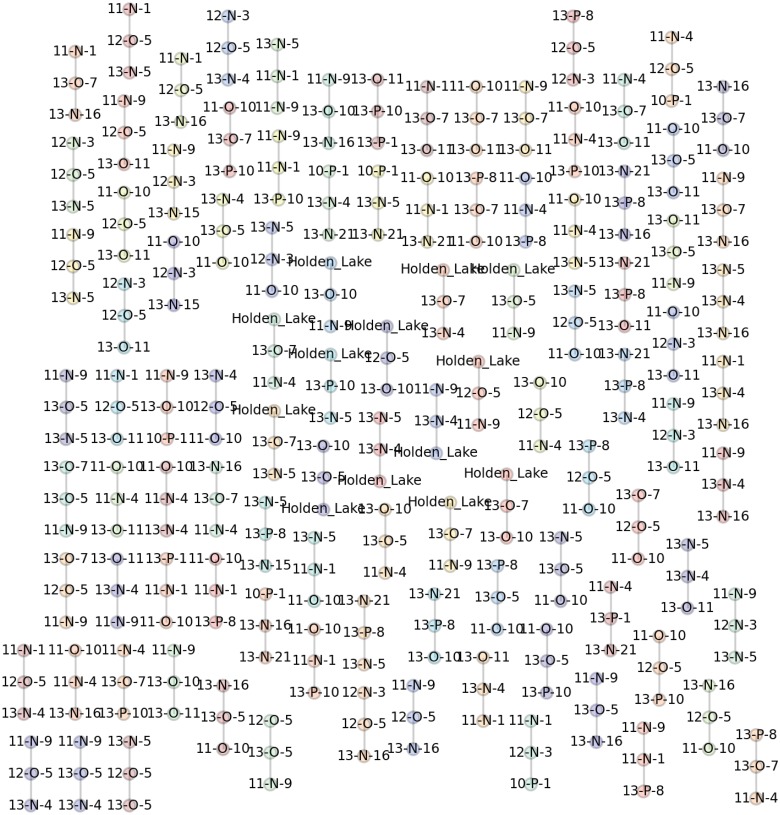
An “atlas” of the 97 valid DFS solutions for the Memphis and St. Francis area assemblages that can be created using the IDSS algorithm, a continuity threshold of 0.30 and *α* = 0.05 confidence intervals for frequency comparisons. The confidence intervals for each assemblage are determined using 1000 bootstrap samples for each pair of assemblages. Note that many assemblages (e.g., 12-O-5) appear in multiple seriations. Also, note that many assemblages are present in more than one solution, which demonstrates the difficulty of understanding the overall pattern of change using the traditional linear representation.

**Fig 12:**
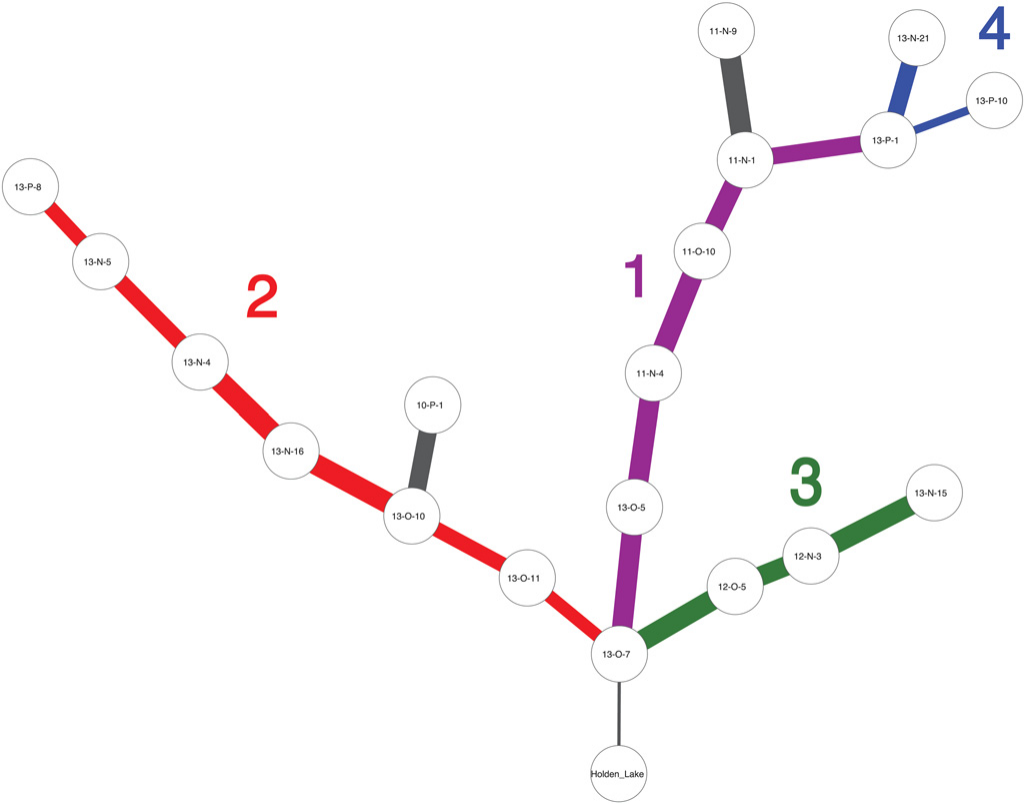
The ‘minmax’ graph produced for the Memphis and St. Francis area assemblages from the 97 valid DFS solutions generated the IDSS algorithm (as shown in Fig 11) using a continuity threshold of 0.30 and *α* = 0.05 confidence intervals for the comparison of frequencies. The “minmax” graph was generated using the procedure described in Fig 6. Significantly, the results show remarkable structure with a series of spatially clustered branches that are formed from overlapping but distinct sets of seriation solutions. Parkin (11-N-1) forms the center of a branch that extends in 3 different directions (to 11-N-9, 13-P-1 and 11-O-10). Assemblages 13-O-7 and 13-O-10 also have this same configuration. 13-O-7 has an extra branch leading to Holden Lake, a presumably earlier deposit. The branches are numbered and colored to correspond with the spatial groups in Fig 13.

**Fig 13:**
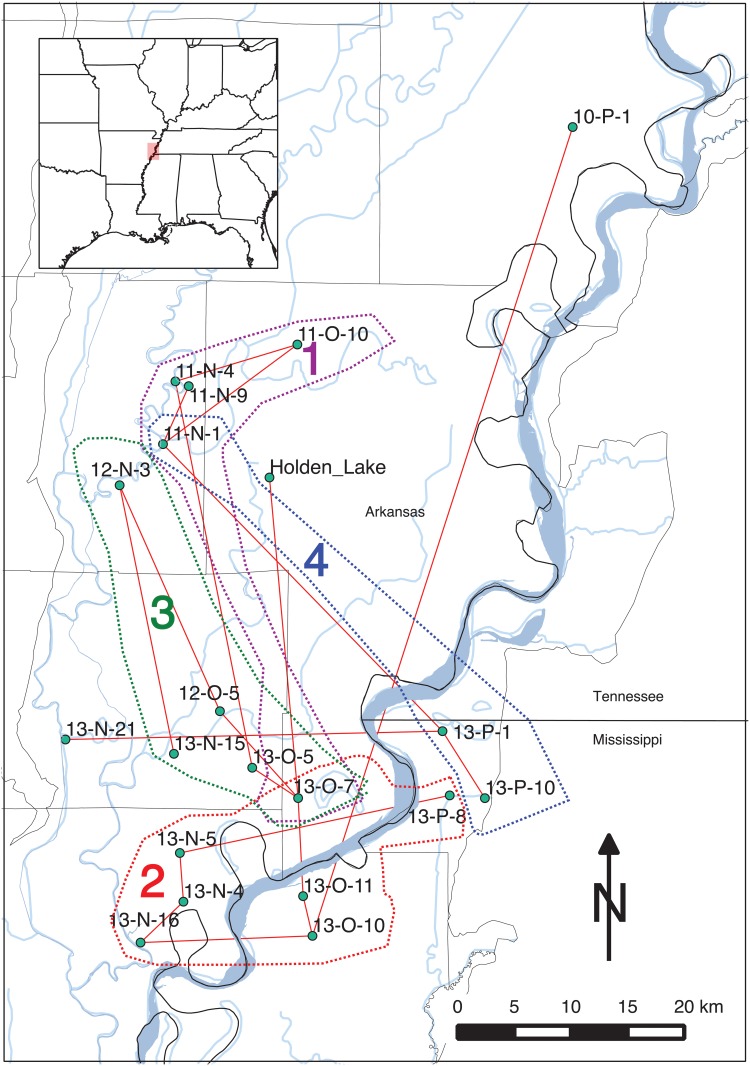
The spatial distribution of the edges of graph shown in Fig 12 and the spatial groups of assemblages. The groups outlines represent the branches of the “minmax” graph depicted in Fig 12. Note that the edges have a strong spatial pattern in that assemblages next to each other are more likely to be paired within seriation solutions than those assemblages that are farther away. A bootstrap assessment of the significance of this spatial pattern shows that *p* = *0.04*. The color of each spatial group corresponds to the major branches in the “minmax” graph in Fig 12.

Using the IDSS analysis we can systematically examine the full set of possible frequency seriation solutions (Figs 11–13). Despite the large number of possible solutions (*N* = 2.56 × 10^18^), iteratively finding the set of 97 possible solutions required less than two seconds of processing due to the fact that the largest possible seriations were composed of only 4 assemblages. No larger sets can be built without introducing violations of unimodality, so the algorithm did not need to continue its search and terminated. Using a confidence interval of *α* = 0.05 allowed us to generate a solution that included all assemblages. Fig 11 clearly shows how the traditional linear ordering breaks down as a visualization mechanism, especially in the presence of many valid solutions. A number of assemblages participate in multiple solutions, and it is impossible to get a sense of the overall nature of the solutions when confronted with many separate orderings. Thus, as described above, we focus here on the graph representation of results (Fig 12).

Fig 12 displays the combination of valid seriation solutions as a “minmax” graph, constructed by combining individual valid solutions and retaining those connecting edges which minimize the total frequency differences between assemblages. Each branch in the graph represents an ordering, which may be temporal, spatial, or a combination of spatiotemporal causes. Most notably, the pattern of the seriation solutions is strongly spatial: assemblages are more likely to be linked to neighbors than others farther away (Fig 13). To assess the statistical significance of the spatial pattern, we resampled the original set of assemblages, and calculated the sum of the frequency distances between the pairs. Doing this 1000 times provided a probability distribution over the clustering of assemblages into groups. In the case of the results shown in Fig 13, we estimated *p* = *0.04* which suggests that the spatial pattern is statistically significant.

The analysis with IDSS shares many of the large scale features of Lipo’s original analysis [83], but there are also significant differences. First, we can now see the continuous nature of the interaction: while there are locally connected sets of assemblages, the seriation solutions all possess interconnections which point to a “nested” interaction structure between communities. In Fig 13, we found 4 groups composed of those sets of assemblages connected to their nearest spatially-local neighbor. Divisions between the identified groups are shown by edges that connect assemblages which minimize intra-group frequency distance. Overall, the IDSS solution reflects the pattern in which assemblages form spatial sets in which are in turn related to each other at higher scales of analysis.

This pattern is exemplified by Group 1 in Fig 13. Group 1 is composed of a single set of assemblages that fall northeast of 11-N-1 (Parkin). Parkin remains a member of more than one seriation solution with branches going to 11-N-9 and another going to a group formed by assemblages 11-O-10 and 11-N-4. Interestingly, on the basis of the IDSS results, Rose Mound (12-N-3) now appears to be more closely related to Group 2 to the south rather than being related to the group with Parkin. This configuration might explain the proximity of the two large deposits so close together. We propose that this set of archaeological deposits were created by separate lineages whose use of the landscape is focused in different directions: Parkin towards the north and Rose Mound to the south. Alternatively, the configuration of assemblage relations may reflect use of the landscape by groups over slightly varying points in time. Further study regarding the relations between these deposits is needed.

Group 2 in Fig 13 includes assemblage 13-P-1, 13-P-10 and 13-N-21 on the east side of the valley. The inclusion of 13-N-21 here can be potentially explained by a series of testable hypotheses: (a) the deposit was created substantially earlier than the other assemblages in the study, (b) the assemblages used in the study are incomplete and lack intermediate assemblages or (c) the composition of the deposit reflects the movement of populations from outside this local community and thus forms a discontinuity. The same set of hypotheses can be built for the relation of 10-P-1, although in this case the lack of additional local assemblages around the deposit is the most likely explanation. Assemblage 13-P-1 shares solutions in the same way in which 11-N-1 does in Group 1.

The assemblages located in the south and southwestern portions of the study area (Group 3 in Fig 13) form a large group in which the likelihood of falling into a solution decreases with distance. The assemblages form two groups (Groups 2 and 3) that overlap at 13-O-7. Like 11-N-1 and 13-P-1, 13-O-7 forms a central node with overlapping seriations, one to the south and one to the north.

The fact that each of the groups of locally interacting assemblages also includes an assemblage that is found in multiple overlapping seriation solutions lends weight to the notion that patterns of interaction reflected in the frequencies of decorated pottery types is informing on the social relations within these communities. Overall, the distance between neighboring communities structures interaction between populations. Interaction, in other words, has a strong “nearest neighbor” quality. A few communities, however, do not follow this pattern and exhibit evidence of greater interaction throughout the region regardless of their frequency distance to other assemblages. This pattern is likely the consequence of hierarchical structure in the interaction among such communities, and potentially represents the beginnings of more complex social organization [80, 83].

Returning briefly to the correspondence analysis from Figs 2 and 3, the clustering of assemblages is roughly similar, but the IDSS results resolve more detail about connections between assemblages. We argue that this detail is available in a deterministic algorithm such as IDSS but not in the correspondence analysis because the transformation of frequency data to similarity coefficients obscures detail, which the traditional frequency seriation model (as embodied here in IDSS) is able to utilize.

## Discussion

DFS has a long history in archaeological research. Indeed, seriation is one of the few unique analytical tools developed entirely within archaeology and its use led to the success of the discipline in the first half of the 20th century. Beginning in the 1960s, the perception grew that DFS was an unsystematic and outdated method of producing chronologies that had been superseded by radiocarbon chronometrics. Radiocarbon dating, however, is not the principal cause for seriation’s demise in recent decades. Despite having broader applicability than just relative dating, the lack of a theoretical rationale and an automated means of generating solutions led investigators to look elsewhere. We suggest that the deficiencies of seriation can be addressed by framing the method in terms of cultural transmission theory and ultimately, evolutionary theory itself. Once integrated into theory and implemented through practical and well-performing algorithms for generating solutions, we argue that seriation has an important place in the archaeological tool kit beyond its former use as a dating method.

The approach presented here certainly does not solve all the problems inherent in the creation of an automated DFS algorithm, but is a step in the right direction. Ultimately, we need a greater understanding of the relations between the structure of the classifications used to categorize and the effect of this structure on seriations. We also need the development of techniques that can handle arbitrarily large sets of assemblages through some combination of careful parsing of valid analytic sets, cluster computing, or clever sorting algorithms. Ideally, we should be able to run DFS analyses on sets of assemblages and then evaluate the results as a function of varying classification strategies, sample sizes and other sources of input. For each source of arbitrary input in the method, we can evaluate the degree to which those choices influence the structure and character of the results. And we need a tighter link between theory and method. For example, what happens if we eliminate the need for unimodality as a sorting criterion? How do assemblages representing different durations affect the structure of outcomes and can we use patterns observed in seriation results to detect duration? Do particular regional models of transmission yield particular patterns in the resulting seriation solutions? Such questions point to new areas of research that are opened up by having an algorithmic means of generating DFS solutions.

The IDSS algorithm reflects an opportunity to achieve some of the promise of seriation as suggested by earlier efforts. Our preliminary results indicates that we can avoid many of the limitations of DFS as traditionally done yet add needed features such as statistical evaluation, automation, and new visual representations to assist in disentangling the roles of time and spatial proximity in solutions. Our example from the Lower Mississippi River Valley illustrates the key features of the approach and demonstrates how IDSS can offer new details about the patterns of prehistoric cultural transmission and inheritance along with statistical assessment of solution quality.

## Supporting Information

S1 Text Pseudocode representation of the IDSS algorithm. (PDF)
