# Supplementary material for: A Theoretically-Sufficient and Computationally-Practical Technique for Deterministic Frequency Seriation
Source: PLoS One. 2015 Apr 29;10(4):e0124942. doi: 10.1371/journal.pone.0124942 (PMC4414518; doi:10.1371/journal.pone.0124942)
Supplement: S1 Text — (PDF) [file pone.0124942.s001.pdf]

Supplementary Information for "A Theoretically-Sufficient And Computationally-Practical Technique For Deterministic Frequency Seriation"

Carl P. Lipo, Mark E. Madsen, and Robert C. Dunnell

March 2015

S1 Text: Pseudo-code representation of the IDSS algorithm

Algorithm 1 Algorithm for IDSS seriation

```
Require: Input file I format:
Tab-delimited text, column 0 contains assemblage name
Remaining columns contain type counts as integers
procedure IDSS(I)
    Read input file I
    Calculate relative frequency of each type
    Calculate max frequency difference between assemblage pairs
    Create list of assemblages A
    for all triplets of assemblages T do
        if using continuity threshold t_c then
            if max frequency difference > t_c for pairs ∈ T then
                Skip triplet
            end if
        end if
        if triplet T is valid given unimodality for all types then
            Store triplet in candidate solutions C
        end if
        R ← assemblages ∉ C                              ▷ Remaining assemblages
        repeat
            for all assemblages a ∈ R do
                if using continuity threshold t_c then
                    if max freq difference > t_c for a and all C then
                        Skip assemblage a for this loop
                    end if
                end if
                if assemblage a + candidate solution c ∈ C then
                    Replace c in C with c + a
                    Remove a from R                      ▷ Grow existing solutions
                end if
            end for
        until R = or loop repeats with no changes
        for all candidate solutions c ∈ C do
            if c is strict subset of another solution in C then
                Remove c from C
            end if
        end for
    end for                                  ▷ C now contains the set of solutions
    Output C in various formats
end procedure
```
